# Identifying TF-MiRNA Regulatory Relationships Using Multiple Features

**DOI:** 10.1371/journal.pone.0125156

**Published:** 2015-04-29

**Authors:** Mingyu Shao, Yanni Sun, Shuigeng Zhou

**Affiliations:** 1 School of Computer Science and Shanghai Key Lab of Intelligent Information Processing, Fudan University, 220 Handan Road, Shanghai 200433, China; 2 Department of Computer Science and Engineering, Michigan State University, 428 S. Shaw Lane, East Lansing, 48824, USA; University of Turin, ITALY

## Abstract

MicroRNAs are known to play important roles in the transcriptional and post-transcriptional regulation of gene expression. While intensive research has been conducted to identify miRNAs and their target genes in various genomes, there is only limited knowledge about how microRNAs are regulated. In this study, we construct a pipeline that can infer the regulatory relationships between transcription factors and microRNAs from ChIP-Seq data with high confidence. In particular, after identifying candidate peaks from ChIP-Seq data, we formulate the inference as a PU learning (learning from only positive and unlabeled examples) problem. Multiple features including the statistical significance of the peaks, the location of the peaks, the transcription factor binding site motifs, and the evolutionary conservation are derived from peaks for training and prediction. To further improve the accuracy of our inference, we also apply a mean reciprocal rank (MRR)-based method to the candidate peaks. We apply our pipeline to infer TF-miRNA regulatory relationships in mouse embryonic stem cells. The experimental results show that our approach provides very specific findings of TF-miRNA regulatory relationships.

## Introduction

MicroRNAs (miRNAs) are small non-coding RNA molecules found in animals, plants, and some viruses and play important roles in transcriptional and post-transcriptional regulation of gene expression [1]. Tremendous efforts have been made for miRNA annotation and regulatory target finding in genomes of various species [2, 3]. However, fewer efforts have been made to understand how miRNAs are regulated. Experimental evidence shows that miRNA genes are usually transcribed by RNA polymerase II (Pol II), like other protein-coding genes [4, 5]. Transcription factors (TFs) first bind to promoter regions of miRNA genes and then recruit PolII to start the transcription of the gene. Usually this step produces long transcripts called primary miRNAs (pri-miRNAs), which will be subsequently cut into one or multiple pre-miRNAs with a hairpin loop structure [4]. After nuclear processing [6], the pre-miRNA is exported into cytoplasm and is further cleaved and forms the mature miRNA [7]. Although miRNAs have been extensively studied, the details of the pri-miRNA regulation, the processing of pri-miRNA into pre-miRNAs, and the final production of the mature miRNAs have not been fully understood. In this work, we focus on the first step of miRNA biogenesis: transcription of pri-miRNAs, which is a fundamental step in production of the mature miRNAs. Like protein-coding genes, the regulation of pri-miRNA transcription is complex, often involving interplay between promoters and regulatory elements such as TFs. A prerequisite for understanding this process is to identify the relationships between miRNAs and TFs. Our goal in this work is to investigate which TFs regulate miRNA transcription.

There has been some existing work focusing on establishing miRNA regulatory network [8–10]. In the network construction, the authors extract TF-miRNA regulatory relationships from existing literature. The quality of the network is partially determined by the accuracy of the interactions between the TFs and their regulated miRNAs. Thus identifying TF-miRNA regulatory relationship is an important step in establishing accurate and comprehensive miRNA regulatory network.

Various methods have been used for identifying TF binding sites. In particular, ChIP-Seq is a method primarily used to analyze how transcription factors and other chromatin-associated proteins interact with DNA and thus regulate gene expression [11, 12]. The workflow of ChIP-Seq consists of two steps: chromatin immunoprecipitation (ChIP) followed by massively parallel DNA sequencing. In the ChIP step, the specific cross-linked DNA-protein complexes are enriched using an antibody against the protein (e.g.,transcription factor) of interest and sheared into DNA fragments by sonication. The antibody quality is crucial to the success of the experiment in this step. In the sequencing step, the ChIP-DNA fragments are sequenced through next-generation sequencing (NGS) platforms. Compared with previous ChIP-chip technology [13], ChIP-Seq offers many advantages such as high base pair resolution, avoiding cross-hybridization between probes and nonspecific targets, no limits on dynamic range of the intensity signal and so forth [14].

However, applying ChIP-Seq data to genome-wide annotation of TF binding sites does not guarantee high accuracy. In the task of peak calling to identify binding sites such as transcription factor binding sites (TFBSs), both steps of ChIP-Seq are error-prone [15]. First, the sequencing errors and biases associated with each sequencing platforms can cause unwanted artifacts. Second, the sample quality, the choice of control data set, and the depth of sequencing can all affect the performance of the experiment [14]. Third, the peak calling algorithms themselves also have limitations. As a result, identifying TFBSs may be subject to high false positive rate, which is the main challenge in the analysis of ChIP-Seq data.

Comparing to identifying TFBSs for protein-coding genes, applying ChIP-Seq data to identify TFs regulating miRNA genes faces one more challenge. The annotation for the promoter regions and transcription start sites (TSSs) of miRNA genes are still incomplete and under study [10, 16]. Thus, the regulatory regions of miRNA genes remains largely unknown in most species. Without knowing the regulatory regions, peak analysis from ChIP-Seq data has to be applied to an estimated promoter region, which may be much larger than the true promoter region, further increasing the false positive rate.

Experimentally inferring TF and miRNA regulatory relationships is difficult to achieve and thus has motivated the development of computational approaches [8–10]. Previous computational methods that used ChIP-Seq data to infer TF and miRNA regulatory networks simply searched ChIP-Seq peaks within certain distances upstream/downstream the TSSs of miRNA genes [10, 17], regardless of the confidence of the peaks.

To address the above mentioned issues, in this study we propose to incorporate multiple features in a machine-learning based classification model for more specific prediction of the TF-miRNA regulatory relationships.

Machine learning algorithms are extensively used in computational biology to give reliable miRNA target predictions [18, 19]. The general approach is that we train one or multiple classifiers using both positive and negative training data and then predict new examples in a test set. However, in many real-world applications, the negative training data is not clearly defined. For instance, as stated in [20], the definite knowledge that a given pair of genes do not interact is typically not available. Similar to gene regulatory networks, TF-miRNA regulatory networks also lack the definite knowledge that a particular TF does not regulate some miRNAs.

With the recent advances of machine learning research, algorithms capable of learning a classifier from positive and unlabeled data only (PU learning) [21] make this situation tractable. There exist several implementations of PU learning for different applications. Of the classifiers used for PU learning, support vector machine (SVM) has gained much attention for its effective classification performance (please refer to [22, 23] for more thorough introduction to classification models and SVM). For instance, Liu *et al.* [24] used the biased SVM to learn text classifiers from positive and unlabeled examples. Mordelet *et al.* [25] divided PU learning into inductive and transductive settings and proposed a bagging SVM method to approach both inductive and transductive PU learning problems. Then Natarajan *et al.* [26] used the transductive setting of bagging SVM to predict gene-disease associations successfully.

Our study focuses on finding positive data from unlabeled examples, which is a typical transductive setting. Here positive data is true TF binding peaks and thus indicates regulatory relationships between TFs and miRNAs. Unlabeled data in our study include all candidate peaks. The bagging SVM method is suitable for the situation that a large pool of unlabeled data is easily available, whereas the size of unlabeled data in our work is similar to that of positive examples, which make the bagging step not suitable. Considering this, we use biased SVM to predict reliable peaks in the regulatory regions of miRNA genes using only positive and unlabeled data, in the transductive way.

In this work, we aim to design an accurate pipeline to infer the TF-miRNA regulatory relationships. The pipeline formulates the inference problem as a classification problem and relies on the following components to reduce the false positive rate. First, we employ a more precise peak calling tool after strict reads quality control. Second, we use multiple features to infer TF-miRNA transcriptional regulatory relationships. The features include: the confidence of peaks detected from ChIP-Seq data, open chromatin regions identified by DNase-Seq, enrichment of TFBS motifs, conservation of peaks, distance of peaks to the miRNA genes, and active miRNAs in the particular cell line (mouse embryonic stem cells in this study). Third, we use a novel machine learning model called *transductive Positive Unlabeled (PU) learning* to infer these relationships. By using PU learning, we can build the classification model with only positive and unlabeled training data, which is a commonly seen scenario for inferring gene regulation relationship. Fourth, we utilize the mean reciprocal rank (MRR) to rank all the chosen peaks and keep only the ones that are also present in the prediction of PU learning as the final result.

We apply our pipeline to infer TF-miRNA regulatory relationships in mouse embryonic stem cells. The experimental results show that our approach provides very specific finding of TF-miRNA regulatory relationships.

## Materials and Methods

**Fig 1:**
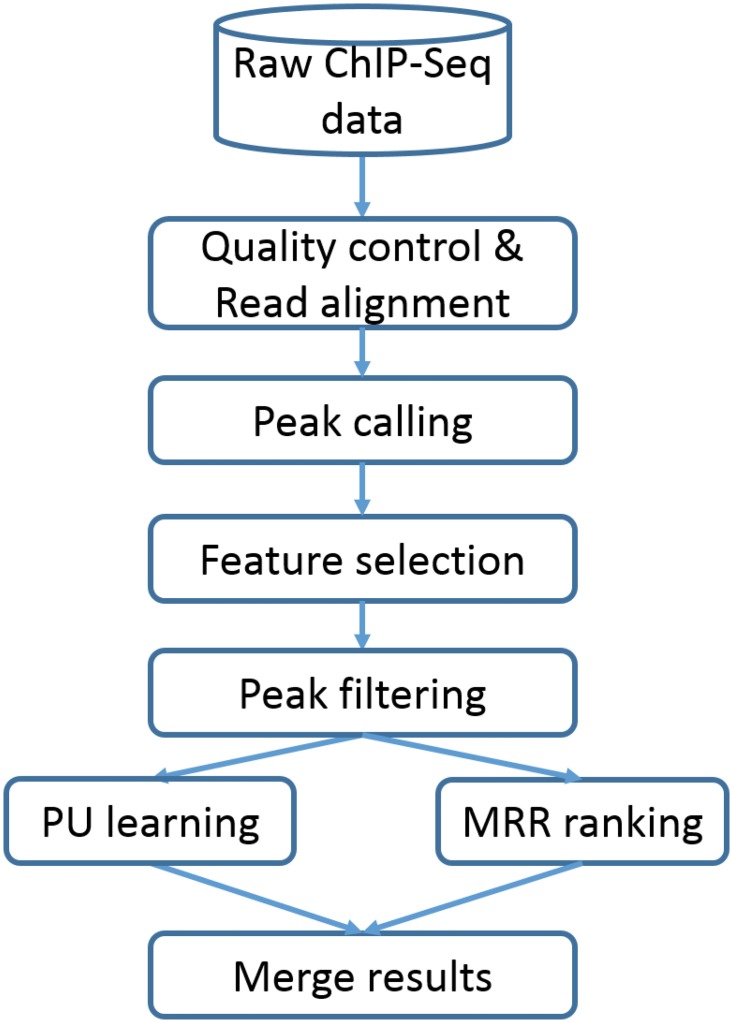
The flowchart of the pipeline. The raw ChIP-Seq data is first preprocessed by filtering low quality reads and aligned to the reference genome. Then peak calling tool is utilized to identify peaks from the alignment. In the next step, multiple features are derived for each candidate peak. The step of peak filtering keeps one peak for one miRNA using the derived features. And then PU learning and MRR ranking are employed and their results are merged to get the final inference.

As miRNAs located within protein coding genes tend to be co-regulated with their parent genes, we focus on identifying TFs that regulate intergenic miRNAs in this work. The major components of our approach are sketched in Fig 1.

Starting with raw ChIP-Seq data, we first remove low quality reads. Then we apply a modified version of MACS to identify peaks within the estimated regulatory regions of the expressed intergenic miRNAs. Usually, multiple peaks exist for each miRNA. However, not every one represents true TFBS. Thus, we derive multiple features for each peak and apply MRR ranking to keep the best peak for each miRNA. Then we apply PU learning and MRR ranking to infer the true TF-miRNA regulatory relationships. Below we describe each component in Fig 1.

### Precise peak calling

In our study, we employed the widely used peak calling tool MACS [27] for enrichment site detection. In ChIP samples, control samples are usually introduced to address the biases of the background read distribution, which is affected by many factors, such as GC content [28] and copy number variation [29]. Therefore, an appropriate estimation of the ChIP/control normalization factor is crucial for enrichment site detection and error rate control [30]. However, MACS is often inaccurate with high bias as it uses the sequencing depth ratio as an estimate of the normalization factor [31]. To tackle this problem, we used a modified version of MACS with the normalization factor calculated separately by NCIS [30], an R package specially developed for the normalization of ChIP-Seq data. Please refer to S2 Text of all the peaks identified by the MACS+NCIS software for all the five TFs considered in our study.

To identify TFs regulating an miRNA, only peaks located within the regulatory regions of mouse intergenic miRNAs (candidate peaks) are kept for further analysis. Below we describe how the regulatory region is determined.

### Determination of regulatory regions for intergenic miRNAs

During miRNA maturation, many regions in the pri-miRNA are degraded quickly, making transcription starting site (TSS) identification of miRNA gene difficult, even with RNA-Seq data available. In addition, identification of miRNA promoter regions is now still under study [10, 16] and the promoter regions of some miRNAs are still unknown. Thus, we need to estimate the regulatory region for intergenic miRNAs. In our study, we define the regulatory regions [32] of intergenic miRNAs as upstream regions with certain distance to the start of intergenic miRNA genes. The lengths of the regions are determined empirically using published studies on miRNA TSS and promoters. As we focus on miRNAs in mouse, we describe how we empirically estimate the size of the regulatory regions for mouse miRNAs. First we investigated the distribution of the distances from the promoter regions to the start of miRNA precursors using recently published results on TSS prediction for miRNAs using TSSvote algorithm in [10]. The farthest distance is approximately 682 kilo bases. Thus we chose 700 kilo bases upstream of the precursors of mouse intergenic miRNA genes as putative regulatory regions. If there are protein-coding genes located in the regulatory regions, we only used the range between the end of the upstream gene and the start of the miRNA precursor as regulatory region. Please see Table A in S1 Text for a complete list of miRNAs studied in our study, together with their genomic positions and the corresponding coordinates of the putative regulatory regions.

Similar to mouse intergenic miRNA regulatory regions, we also define the regulatory regions for human miRNAs in order to extract relevant features (see the “Feature Extraction” Section). The determination process is basically the same. We chose 1000 kilo bases upstream of the start of the miRNA precursors as putative regulatory regions based on the distance distribution obtained from [10].

We use large putative regulatory regions to identify TFBS that are distant to miRNA genes. It is possible that some false TFBS will be included. Thus we employ multiple features (described below) to control the false positive rate.

### Feature extraction

Peak calling can return a number of peaks located within the estimated regulatory regions. We extract multiple features for each peak to identify true peaks caused by TF binding. These features are used in our classification model for true TFBS prediction.

#### Statistical significance of peaks

The statistical characteristics generated by peak calling tool show the statistical significance of the peaks. Correspondingly, we extract the number of tags, the p-value, and the fold enrichment of the peaks located within the regulatory regions. They represent the number of reads in a peak region, the significance of the peak region against the local background region, and the fold enrichment for this region compared to the expectation from Poisson distribution with local lambda [27] respectively.

#### The location of peaks

As mentioned in [15], most TFs require an open chromatin region to stably bind to their DNA targets. Hence, if a candidate peak is located in the open chromatin region, this peak is more likely to contain a TFBS. DNase-Seq [33], one of the assays developed to detect open chromatin, can be utilized to check whether a candidate peak is located in open chromatin regions. In our study, the status of a peak (within the open chromatin region or not) is viewed as a feature for prediction. We downloaded the mouse ES cell DNase-Seq peaks from UCSC genome browser [34] (Accession number: wgEncodeEM003417). As there are two replicates in the DNase-Seq data, we utilized MAnorm [35] to obtain concordant peaks of the two replicates with parameters provided in the tutorial. Then each candidate miRNA-related peak is examined whether it has at least one base overlap with the DNase-Seq peaks.

Generally, transcription factors are in the proximity of the genes that they regulate. Thus the distance from a peak summit to the start of miRNA gene is also adopted as a location feature for prediction.

#### TFBS motifs

The motif binding score within a peak region is calculated and used as a feature. The motifs are usually modeled by Position Weight Matrices (PWMs). We can search a sequence for possible binding sites of a particular TF using its PWM by calculating a motif score (log-odds score). Sequences with higher motif scores are more likely to contain real TFBSs. In our study, we download the PWMs for the five TFs studied in this work from the JASPAR databases [36]. We extract the 200-bp region centered upon the summit of each peak to ensure the confidence of the peak and calculate the log-odds score at each nucleotide position of the region. The maximum of the log-odds scores of the region is regarded as the motif score of this peak and used as a feature for further prediction.

#### Evolutionary conservation

The conservation between species can provide biological support for finding reliable TFBS. If a peak is located within the regulatory regions of both a miRNA gene and its homologous miRNA gene, this peak is very likely to be a real TFBS. Specifically, we utilize the liftover tool [37] to map the candidate miRNA-related peaks to human genome. Then we decide whether these peaks located within the regulatory regions of human miRNAs. For peaks located within both the human and the mouse miRNA regulatory regions, we further investigate whether the mouse miRNA and the human miRNA are homologous. The regulatory relationships between TFs and miRNAs are considered to be conserved only if the candidate miRNA-related peaks are located in the regulatory regions of homologous miRNAs. All genomic coordinates of other mouse genome assemblies are lifted to that of the latest mouse assembly GRCm38 (UCSC version mm10) while all genomic coordinates of other human genome assemblies are lift to that of the latest human assembly GRCh37 (USCS version hg19) using the liftOver utility from UCSC (downloaded from http://hgdownload.cse.ucsc.edu/admin/exe/) with default parameters.

Since the features utilized in our research have values at dramatically different scales (please refer to S3 Text for the determination of the feature values), we use the following formula to normalize these features to the range of [0, 1]:

$$\begin{array}{c} x_{i}=\frac{x_{i}-x_{min,i}}{x_{max,i}-x_{min,i}}, \end{array}$$ (1)

where *x*_*max*,*i*_, *x*_*min*,*i*_ denote the maximum and the minimum of feature *i*, respectively.

### Mean reciprocal rank (MRR) calculation

MRR [38] was initially used in question answering systems [39]. Later, it was used to rank protein-protein interaction (PPI) network models with multiple topological features [40]. MRR is defined as:

$$\begin{array}{c} MRR=\frac{1}{\left|N\right|}\sum_{i=1}^{\left|N\right|}\frac{1}{rank_{i}}, \end{array}$$ (2)

where *N* denotes the number of features, and *rank*_*i*_ means the rank of the *i*th feature.

Our situation is similar to the ranking of PPI network models. For most intergenic miRNAs, we identified multiple peaks located in the same regulatory region that are likely to contain potential TFBS, and each peak has several “features” showing its statistical significance or biological significance. Thus, the MRR value indicates the integrated significance of a peak. For each miRNA, we chose the peak with the highest MRR value for further analysis. In the case of two peaks having the same MRR value, we pick the peak closer to the corresponding miRNA.

Besides peak filtering using MRR, we also employed the MRR value to rank all the miRNAs for a specific TF. That is, after choosing only one peak for an miRNA, we calculated the MRR values of the peaks for all miRNAs and then ranked these miRNAs based on these MRR values.

### PU learning

In our work, multiple candidate peaks are identified for each TF, and each of these candidate peaks have multiple features (please see the Feature Extraction Section). Since these peaks located within the regulatory regions of miRNAs, we can represent the miRNAs as corresponding peaks. The goal of PU learning in our work thus is to predict whether the miRNA is regulated by this TF.

Specifically, for each TF, we first obtain some known TF-miRNA relationships. For these miRNAs, we used the corresponding peaks as positive training examples, and the rest peaks are treated as unlabeled examples.

We utilized the SVMlight software to accomplish the PU learning task. SVMlight is an implementation of SVM introduced in [23]. It contains an algorithm for training transductive SVMs and a detailed description of the algorithm can be found in [41]. Specifically, SVMlight uses the transductive parameter *p* to control the fraction of unlabeled examples to be classified into the positive class. By setting different values of *p*, we can get different prediction results. Generally, the larger of the value of *p*, the more examples in the unlabeled data will be predicted as positive. In our experiment setting, we varied the value of *p* from 0.05 to 0.95 with a step size of 0.05.

### Results merging

**Fig 2:**
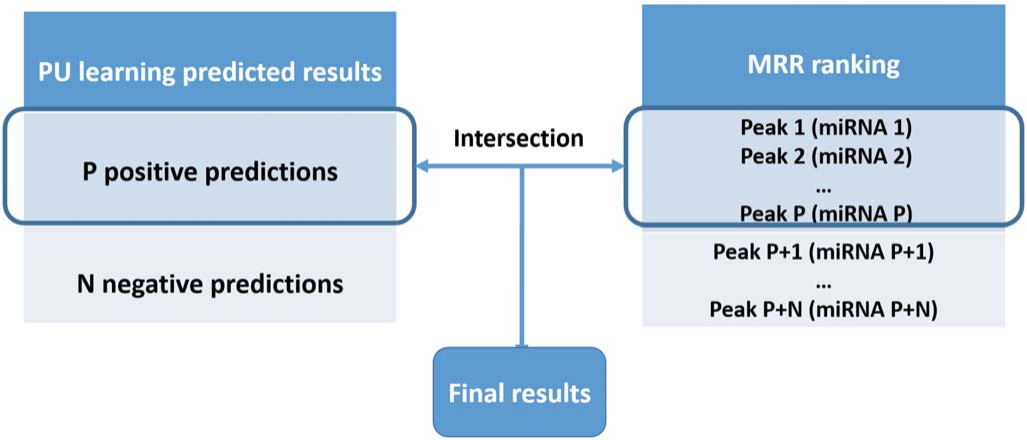
The illustration of the merge step. We got *P* positive predictions from PU learning and a list of ranked candidate peaks by MRR ranking. Then we took the top *P* peaks from the list and intersected them with the *P* positive predictions from PU learning. The intersection is the final result.

After we obtained the predicted results of PU learning and the ranking of all candidate peaks by their MRR values, we merged the results. The merging step is illustrated in Fig 2.

## Results

We applied our pipeline to study the regulatory relationships for intergenic miRNAs in mouse embryonic stem cell considering the importance of miRNAs in embryonic stem cells [42]. There are 304 intergenic miRNAs available in miRBase [2] in total, and 123 of these miRNAs were detected expressed on at least one sequencing platform in mouse embryonic stem cell according to a recent survey [43]. Therefore, we focus on studying TF-miRNA regulatory relationships for these 123 expressed intergenic miRNAs.

Based on the availability of the features, such as DNase-Seq data, we focus on five TFs that have all the mentioned features available. They are Oct4, Sox2, Klf4, Esrrb, and Tcf3. We downloaded the mouse embryonic stem cell (mESC) ChIP-Seq data sets from the Sequence Read Archive (http://www.ncbi.nlm.nih.gov/sra/): Oct4 (SRX000546 [44]), Sox2 (SRX000549 [44]), Klf4 (SRX000544 [44]), Esrrb (SRX000542 [44]), Tcf3 (SRX003870 [42], SRX003871 [42]), mESC input (SRX000543 [44], SRX003873 [42], SRX003872 [42]).

The raw data was converted into FASTQ files using the fastq-dump command of SRA Toolkit and the reads were preprocessed using the fastq_quality_trimmer command of FASTX Toolkit (-t 20 -l 16) [45]. Reads passing the quality control were mapped to the mouse genome assembly GRCm38 (UCSC version: mm10) with Bowtie [46] allowing two mismatches. Only reads with unique alignment were kept.

Next, after precise peak calling and feature extraction, we employed the SVMlight software to predict TF-miRNA regulatory relationships from these candidate peaks (please see the Materials and Methods Section for details).

### Evaluating the performance of PU learning using different training data sets

First, we designed three different training data sets to investigate how the training examples and the value of parameter *p* affect the prediction performance. We used the ChIP-Seq data set of transcription factor Oct4 to carry out this investigation. The positive training data can include peaks derived from two types of TF-gene regulatory relationships. One is TFs that regulate protein-coding genes. The other is TFs regulating miRNA genes. Using one type of peaks or the combined peaks, we design three kinds of positive training data. Specifically, we used only miRNA related positive samples (11 samples), only protein-coding gene related positive samples (57 samples), and both miRNA and protein-coding gene related positive samples (68 samples) as positive training set respectively to train the classifier. From each positive training set, we randomly selected 10%, 30%, 50%, 70%, 90% of the positive examples as unlabeled examples, respectively. Then for each training set, we varied the value of parameter *p* from 0.05 to 0.95 with a step size of 0.05.

**Fig 3:**
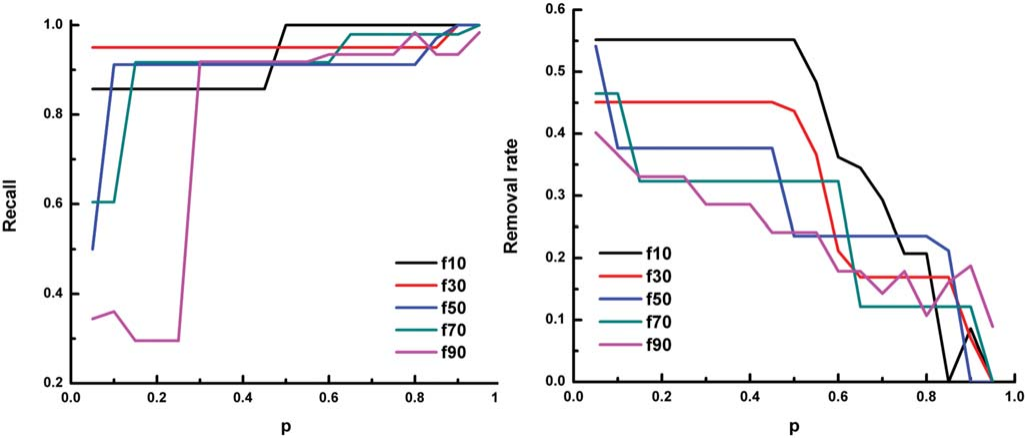
The recall and removal rate of prediction on Oct4-miRNA relationships using known regulation between Oct4 and protein-coding genes or miRNA genes. In each panel, the x-axis denotes the parameter *p* of SVMlight. The y-axis denotes the recall (left panel) and the removal rate (right panel) of the prediction, respectively. f10 means 10% of the known positive examples are put into the unlabeled data,and f30, ⋯, f90 mean 30%, ⋯, 90% of the known positive examples are put into the unlabeled data, respectively.

We use recall and removal rate to evaluate the prediction performance of SVMlight throughout the study. Recall is defined as the fraction of predicted positive examples in the set of known positive examples. Removal rate means the fraction of examples predicted as negative in the unlabelled data. A sensitive and specific classifier should achieve a high recall rate while being able to remove a large number of negative peaks (i.e. high removal rate). Fig 3 shows the prediction performance using the positive training data that includes the peaks derived from known regulatory relationships between Oct4 and protein-coding genes or miRNA genes.

From the figure, we can see that the value of parameter *p* has a significant effect on the prediction performance: with the increase of *p* value, the recall increases while the removal rate decreases. The fraction of selected positives as unlabeled data also affects the prediction performance. Generally, with more positives selected as unlabeled data, the recall of the prediction decreases while the removal rate decreases more quickly with the increase of the *p* value. Thus, in our following experiments (see Section “Inferring TF-miRNA regulatory relationships”), we used all known positive samples as positive training data set.

**Fig 4:**
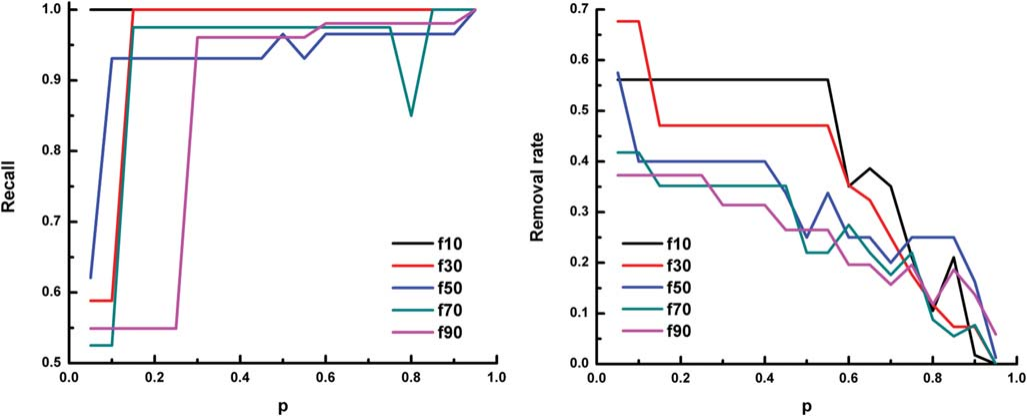
The recall and removal rate of prediction on Oct4-miRNA relationships using only known regulation between Oct4 and protein-coding genes. In each panel, the x-axis denotes the parameter *p* of SVMlight. The y-axis denotes the recall (left panel) and the removal rate (right panel) of the prediction, respectively. f10 means 10% of the known positive examples are put into the unlabeled data,and f30, ⋯, f90 mean 30%, ⋯, 90% of the know positive examples are put into the unlabeled data, respectively.

**Fig 5:**
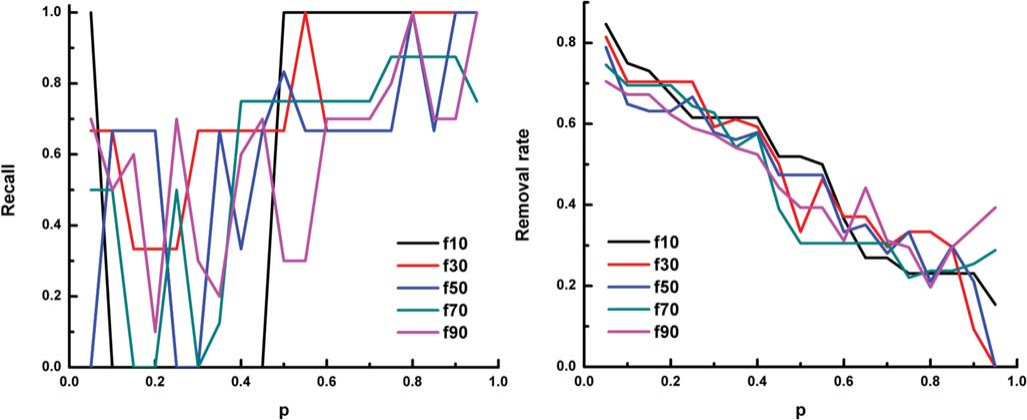
The recall and removal rate of prediction on Oct4-miRNA relationships using only known regulation between Oct4 and miRNA genes. In each panel, the x-axis denotes the parameter *p* of SVMlight. The y-axis denotes the recall (left panel) and the removal rate (right panel) of the prediction, respectively. f10 means 10% of the known positive examples are put into the unlabeled data,and f30, ⋯, f90 mean 30%, ⋯, 90% of the know positive examples are put into the unlabeled data, respectively.

The performance of using only known regulation between Oct4 and protein-coding genes as the positive training data (Fig 4) is similar to the above result, especially when a small fraction of positives are selected as unlabeled data. However, as the known TF-miRNA relationships are too limited, the performance of using only known regulation between Oct4 and miRNA genes as the positive training data (Fig 5) is not satisfactory. This result indicates that we can still predict the regulatory relationships for miRNA genes when only protein-coding gene related peaks are available. Thus, for transcription factors that do not have known regulated miRNAs (e.g. Esrrb and Klf4), we used protein-coding gene related peaks only as positive samples in the following experiments.

### Inferring TF-miRNA regulatory relationships

We employed ChIP-Seq data of more TFs to investigate whether they regulate miRNAs in the mouse embryonic stem cell. In these experiments, we used all known regulatory relationships as positive training data to predict the unlabeled data.

**Fig 6:**
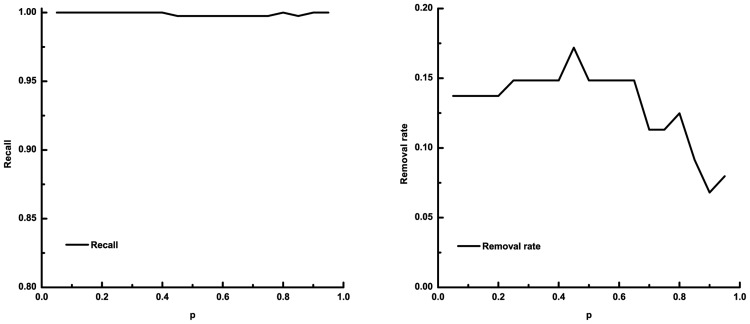
The recall and removal rate of prediction on Esrrb-miRNA relationships using protein-coding gene related positive data sets of transcription factor Esrrb and five-fold cross validation. In each panel, the x-axis denotes the parameter *p* of SVMlight, it ranges from 0.05 to 0.95 with a step size of 0.05. The y-axis denotes the recall (left panel) and the removal rate (right panel) of the prediction, respectively.

Fig 6 demonstrates the recall and the removal rate with different *p* values using five-fold cross validation for the Esrrb transcription factor (the results for the other four TFs can refer to Fig. A–D in S1 Text). We can see from the left panel of the figure that the recall is generally high with different *p* values. In order to further determine the performance of the classifier, we also computed the removal rate of unlabeled data. From the right panel, we can see that quite a portion of unlabeled data are predicted as negatives. This indicates that the classifier can retrieve almost all positives while still removing possible negatives.

From the results of our prediction, we find that although different values of *p* result in different predictions, some miRNAs are always predicted as positives regardless of the value of *p*. We kept those miRNAs as the predicted result only in order to minimize false positive rate.

**Fig 7:**
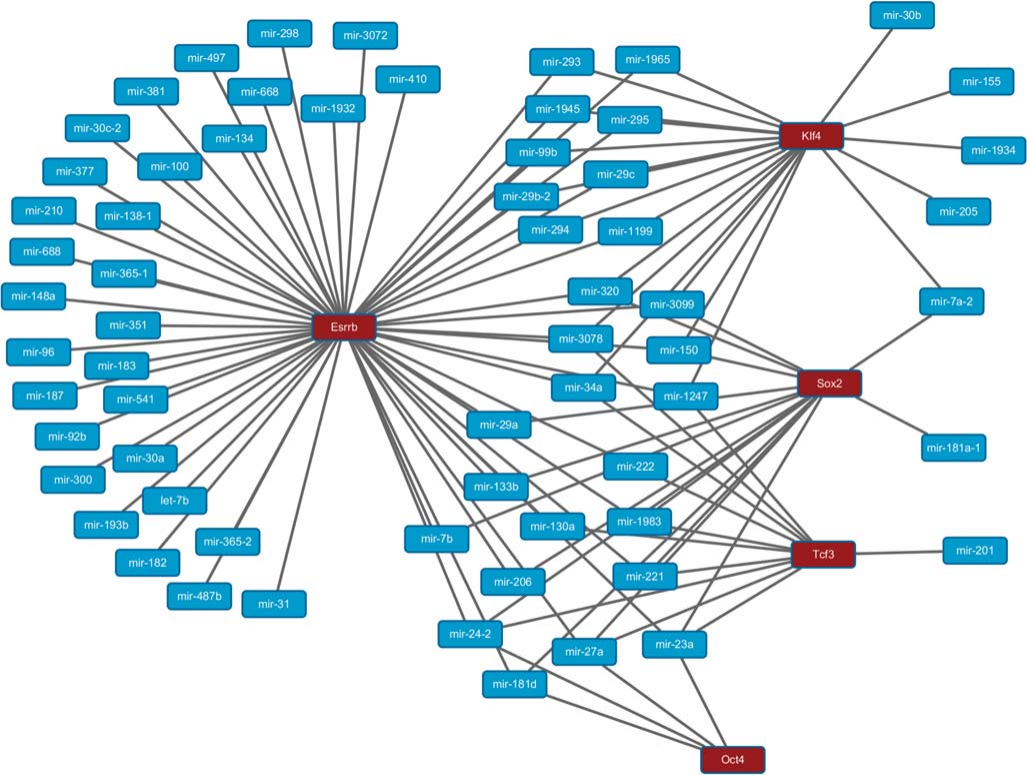
The inferred regulatory relationships for the five TFs studied in our work. The red boxes indicate the five TFs and the blue boxes indicate the miRNAs. The lines between the red and blue boxes indicate the regulatory relationships between the two entities.

Meanwhile, we also ranked all the peaks chosen for the miRNAs by their MRR values and kept the peaks that also present in the prediction of SVMlight as the final result. Fig 7 shows the inferred regulatory relationships of the five TFs studied in our work. The final prediction result is also provided in Table B in S1 Text.

### Evaluation of regulatory relationships

We compared part of the regulatory relationships inferred by our pipeline with TF-miRNA relationships in the ChIPBase database [17]. As there are four overlapping TFs between the ChIPBase database and our study, we compared regulatory relationships of these four TFs and downloaded the identified regulatory relationships between TFs and miRNAs from ChIPBase as ChIPBase relationships.

We only compared the regulatory relationships with those derived from the same ChIP-Seq datasets as ours. Since we only adopted expressed intergenic miRNAs in our study, we filtered the miRNAs in the obtained ChIPBase relationships and kept only those expressed in mouse embryonic stem cell [43].

Table 1 show the comparison results of ChIPBase relationships and our predicted relationships. From this table, we can see that the overlap between these two sets is not so large. There are two reasons for this discrepancy. One is that we identified peaks using the raw ChIP-Seq data rather than directly using the peaks provided in the datasets (hereafter “original peaks” for convenience) as ChIPBase. Different from the original peak calling procedure, we filtered out low quality reads before aligning them to the genome and used a modified version of MACS which can generate many more peaks. The former operation will miss some peaks in the original peaks while the latter operation will find more peaks than the original peaks (Table 2). The other reason is that we used much larger regulatory regions than ChIPBase. Specifically, we used 700 kilo bases upstream of each miRNA gene as the regulatory region while ChIPBase uses 30 kilo bases (maximum) upstream and 10 kilo bases (maximum) downstream of each miRNA gene as the regulatory domain/region.

**Table 1:** Comparison of our final result with ChIPBase relationships of the four TFs: Esrrb, Klf4, Oct4 and Sox2.

TF	ChIPBase relationships	Predicted relationships	Overlapped relationships
Esrrb	40	57	26
Klf4	27	20	7
Oct4	31	4	4
Sox2	9	15	1

**Table 2:** Comparison of peaks found by MACS+NCIS used in our study and the original peaks published with Oct4 and Sox2 ChIP-Seq datasets.

TF	Original peaks only	NCIS peaks only	Overlap
Oct4_Without QC	2	100	46
Oct4_With QC_16	3	83	45
Sox2_Without QC	0	82	52
Sox2_With QC_16	6	73	46

**With QC_16** means before mapping to the genome, low quality reads and reads having a length of less than 16 nt are removed using quality control tool FASTX-Toolkit [45].

**Without QC** means no quality control tool is applied to the reads before mapping them to the genome.

Besides ChIPBase, we also searched for the literature that focuses on TF-miRNA regulatory relationships. Since we focus on the five TFs and the expressed intergenic miRNAs in mouse embryonic stem cell, which has a rather restricted condition on the TFs, miRNAs, and the cell lines, we did not find much appropriate TF-miRNA regulatory relationships in the literature. However, we succeeded predicting the one regulatory relationship (i.e., Esrrb–mmu-let-7d) in CircuitsDB [47] that matches our miRNAs and TFs.

## Discussion

In this study, we used ChIP-Seq data to predict the regulatory relationships between TFs and miRNA genes. We first obtained significant peaks (candidate TFBSs) located in miRNA regulatory regions from ChIP-Seq data, and then screen these peaks using carefully chosen features. Specifically, we employed transductive PU learning to predict whether these peaks are true TFBSs, and then analyze the TF-miRNA regulatory relationships based on the predicted TFBSs.

The main disadvantage of ChIP-Seq assay is the high false positive rate in identifying binding sites. ChIP-exo [48] assay, a modification to the standard ChIP-Seq protocol, can identify binding sites more precisely with a lower background signal [15]. It would be a good substitute of ChIP-Seq method to detect binding sites in our research if sufficient data is available.

With regard to selecting the features for the peaks, we considered the following aspects: 1), the characteristics of peak-calling, such as the number of tags, enrichment, p-value and the distance from the peaks to the corresponding miRNA gene TSSs; 2), the characteristics of the sequence, i.e. the motif score; 3), the position of the peaks. We examined whether the peaks are located in open chromatin region using DNase-Seq data. and 4), the evolutionary conservation information. We checked whether a peak located within the regulatory region of a mouse miRNA gene could be mapped to the regulatory region of the homologous miRNA gene in human.

One problem during feature determination is the incompleteness of various data. For instance, the number of TFs that we considered in our study is largely limited due to the availability of the corresponding DNase-Seq datasets. Meanwhile, some useful features, such as histone modifications, which have important roles in transcriptional regulation [49], have to be abandoned owing to lack of corresponding datasets. The availability of more complete datasets will enable us to utilize more useful features and thus enhance the performance of predictions.

We utilized SVMlight package to predict the relationships and used the parameter *p* to adjust the fraction of predicted positive data in unlabeled data. Since the actual fraction of positives in the unlabeled data is unknown, we varied the value of *p* to obtain different predictions. We kept only the peaks that were predicted positives under all *p* values. Therefore, our prediction has higher confidence than the one obtained using a single *p* value.

## Supporting Information

S1 Text Supporting Figures and Tables. **Fig. A. The recall and removal rate of prediction on Klf4-miRNA relationships using protein-coding gene related positive data sets of transcription factor Klf4 and five-fold cross validation**. In each panel, the x-axis denotes the parameter *p* of SVMlight. It ranges from 0.05 to 0.95 with a step size of 0.05. The y-axis denotes the recall (left panel) and the removal rate (right panel) of the prediction, respectively.
**Fig. B. The recall and removal rate of prediction on Oct4-miRNA relationships using protein-coding gene and miRNA gene related positive data sets of transcription factor Oct4 and five-fold cross validation**. In each panel, the x-axis denotes the parameter *p* of SVMlight. It ranges from 0.05 to 0.95 with a step size of 0.05. The y-axis denotes the recall (left panel) and the removal rate (right panel) of the prediction, respectively.
**Fig. C. The recall and removal rate of prediction on Sox2-miRNA relationships using protein-coding gene and miRNA gene related positive data sets of transcription factor Sox2 and five-fold cross validation**. In each panel, the x-axis denotes the parameter *p* of SVMlight. It ranges from 0.05 to 0.95 with a step size of 0.05. The y-axis denotes the recall (left panel) and the removal rate (right panel) of the prediction, respectively.
**Fig. D. The recall and removal rate of prediction on Tcf3-miRNA relationships using protein-coding gene and miRNA gene related positive data sets of transcription factor Tcf3 and five-fold cross validation**. In each panel, the x-axis denotes the parameter *p* of SVMlight. It ranges from 0.05 to 0.95 with a step size of 0.05. The y-axis denotes the recall (left panel) and the removal rate (right panel) of the prediction, respectively.
**Table A. The genomic coordinates of the miRNA genes and their putative regulatory regions used in our study**. Here Chr in the second column means the chromosome that the miRNA gene located in. G_start_ and G_end_ mean the start and end coordinates of the miRNA gene, respectively. R_start_ and R_end_ mean the start and end coordinates of the putative regulatory regions of this miRNA gene, respectively. Please note that the start coordinate is always smaller than the end coordinate in this table, regardless of which strand the miRNA gene is located in.
**Table B. Final prediction result for the five TFs: Esrrb, Klf4, Oct4, Sox2, and Tcf3**. The number in the parentheses behind the TF indicates the count of miRNAs regulated by this particular TF.
(PDF)

S2 Text Peaks identified by the MACS+NCIS software. (ZIP)

S3 Text Description of the feature values. (PDF)

## References

[1] Chen K, Rajewsky N. The evolution of gene regulation by transcription factors and microRNAs. Nature Reviews Genetics. 2007;8(2):93–103. 10.1038/nrg1990 17230196

[2] Griffiths-Jones S, Saini HK, van Dongen S, Enright AJ. miRBase: tools for microRNA genomics. Nucleic Acids Research. 2008;36(suppl 1):D154–D158. 10.1093/nar/gkm952 17991681 PMC2238936

[3] Bartel DP. MicroRNAs: target recognition and regulatory functions. Cell. 2009;136(2):215–233. 10.1016/j.cell.2009.01.002 19167326 PMC3794896

[4] Lee Y, Kim M, Han J, Yeom KH, Lee S, Baek SH, et al MicroRNA genes are transcribed by RNA polymerase II. The EMBO Journal. 2004;23(20):4051–4060. 10.1038/sj.emboj.7600385 15372072 PMC524334

[5] Zhou X, Ruan J, Wang G, Zhang W. Characterization and identification of microRNA core promoters in four model species. PLoS Computational Biology. 2007;3(3):e37 10.1371/journal.pcbi.0030037 17352530 PMC1817659

[6] Murchison EP, Hannon GJ. miRNAs on the move: miRNA biogenesis and the RNAi machinery. Current Opinion in Cell Biology. 2004;16(3):223–229. 10.1016/j.ceb.2004.04.003 15145345

[7] Ji X. The mechanism of RNase III action: how dicer dices In: RNA Interference. Berlin Heidelberg: Springer; 2008 p. 99–116. 10.1007/978-3-540-75157-1_5 18268841

[8] Bisognin A, Sales G, Coppe A, Bortoluzzi S, Romualdi C. MAGIA2: from miRNA and genes expression data integrative analysis to microRNA-transcription factor mixed regulatory circuits (2012 update). Nucleic Acids Research. 2012;40(W1):W13–W21. 10.1093/nar/gks460 22618880 PMC3394337

[9] Le Bechec A, Portales-Casamar E, Vetter G, Moes M, Zindy PJ, Saumet A, et al MIR@NT@N: a framework integrating transcription factors, microRNAs and their targets to identify sub-network motifs in a meta-regulation network model. BMC Bioinformatics. 2011;12(1):67 10.1186/1471-2105-12-67 21375730 PMC3061897

[10] Chang LW, Viader A, Varghese N, Payton JE, Milbrandt J, Nagarajan R. An integrated approach to characterize transcription factor and microRNA regulatory networks involved in Schwann cell response to peripheral nerve injury. BMC Genomics. 2013;14(1):84 10.1186/1471-2164-14-84 23387820 PMC3599357

[11] Johnson DS, Mortazavi A, Myers RM, Wold B. Genome-wide mapping of in vivo protein-DNA interactions. Science. 2007;316(5830):1497–1502. 10.1126/science.1141319 17540862

[12] Robertson G, Hirst M, Bainbridge M, Bilenky M, Zhao Y, Zeng T, et al Genome-wide profiles of STAT1 DNA association using chromatin immunoprecipitation and massively parallel sequencing. Nature Methods. 2007;4(8):651–657. 10.1038/nmeth1068 17558387

[13] Aparicio O, Geisberg JV, Sekinger E, Yang A, Moqtaderi Z, Struhl K. Chromatin immunoprecipitation for determining the association of proteins with specific genomic sequences in vivo. Current Protocols in Molecular Biology. 2005;p. 21–3. 10.1002/0471142727.mb2103s69 18265358

[14] Park PJ. ChIP-Seq: advantages and challenges of a maturing technology. Nature Reviews Genetics. 2009;10(10):669–680. 10.1038/nrg2641 19736561 PMC3191340

[15] Furey TS. ChIP-Seq and beyond: new and improved methodologies to detect and characterize protein-DNA interactions. Nature Reviews Genetics. 2012;13(12):840–852. 10.1038/nrg3306 23090257 PMC3591838

[16] Corcoran DL, Pandit KV, Gordon B, Bhattacharjee A, Kaminski N, Benos PV. Features of mammalian microRNA promoters emerge from polymerase II chromatin immunoprecipitation data. PLoS One. 2009;4(4):e5279 10.1371/journal.pone.0005279 19390574 PMC2668758

[17] Yang JH, Li JH, Jiang S, Zhou H, Qu LH. ChIPBase: a database for decoding the transcriptional regulation of long non-coding RNA and microRNA genes from ChIP-Seq data. Nucleic Acids Research. 2013;41(D1):D177–D187. 10.1093/nar/gks1060 23161675 PMC3531181

[18] Mendoza MR, da Fonseca GC, Loss-Morais G, Alves R, Margis R, Bazzan AL. RFMirTarget: Predicting Human MicroRNA Target Genes with a Random Forest Classifier. PloS One. 2013;8(7):e70153 10.1371/journal.pone.0070153 23922946 PMC3724815

[19] Kurubanjerdjit N, Huang CH, Lee YI, Tsai JJ, Ng KL. Prediction of microRNA-regulated protein interaction pathways in Arabidopsis using machine learning algorithms. Computers in Biology and Medicine. 2013;. 10.1016/j.compbiomed.2013.08.010 24209909

[20] Cerulo L, Elkan C, Ceccarelli M. Learning gene regulatory networks from only positive and unlabeled data. BMC Bioinformatics. 2010;11(1):228 10.1186/1471-2105-11-228 20444264 PMC2887423

[21] Elkan C, Noto K. Learning classifiers from only positive and unlabeled data. In: Proceedings of the 14th ACM SIGKDD international conference on Knowledge discovery and data mining. ACM; 2008. p. 213–220.

[22] Mitchell TM. Machine Learning. 1st ed. New York, NY, USA: McGraw-Hill, Inc.; 1997.

[23] Vapnik VN. The Nature of Statistical Learning Theory. New York, NY, USA: Springer-Verlag New York, Inc.; 1995.

[24] Liu B, Dai Y, Li X, Lee WS, Yu PS. Building Text Classifiers using Positive and Unlabeled Examples. In: In Proc. of the ICDM03; 2003 p. 179–188.

[25] Mordelet F, Vert JP. A bagging SVM to learn from positive and unlabeled examples. arXiv preprint arXiv:10100772. 2010.

[26] Natarajan N, Blom UM, Tewari A, Woods JO, Dhillon IS, Marcotte EM. Predicting gene-disease associations using multiple species data. Department of Computer Science, University of Texas at Austin, Tech Rep TR-11-37 2011;.

[27] Zhang Y, Liu T, Meyer CA, Eeckhoute J, Johnson DS, Bernstein BE, et al Model-based analysis of ChIP-Seq (MACS). Genome Biology. 2008;9(9):R137 10.1186/gb-2008-9-9-r137 18798982 PMC2592715

[28] Dohm JC, Lottaz C, Borodina T, Himmelbauer H. Substantial biases in ultra-short read data sets from high-throughput DNA sequencing. Nucleic Acids Research. 2008;36(16):e105–e105. 10.1093/nar/gkn425 18660515 PMC2532726

[29] Vega VB, Cheung E, Palanisamy N, Sung WK. Inherent signals in sequencing-based chromatin-immunoprecipitation control libraries. PLoS One. 2009;4(4):e5241 10.1371/journal.pone.0005241 19367334 PMC2666154

[30] Liang K, Keleş S. Normalization of ChIP-Seq data with control. BMC Bioinformatics. 2012;13(1):199 10.1186/1471-2105-13-199 22883957 PMC3475056

[31] Sun G, Chung D, Liang K, Keleş S. Statistical Analysis of ChIP-Seq Data with MOSAiCS In: Deep Sequencing Data Analysis. Berlin Heidelberg: Springer; 2013 p. 193–212. 10.1007/978-1-62703-514-9_12 23872977

[32] McLean CY, Bristor D, Hiller M, Clarke SL, Schaar BT, Lowe CB, et al GREAT improves functional interpretation of cis-regulatory regions. Nature Biotechnology. 2010;28(5):495–501. 10.1038/nbt.1630 20436461 PMC4840234

[33] Boyle AP, Davis S, Shulha HP, Meltzer P, Margulies EH, Weng Z, et al High-resolution mapping and characterization of open chromatin across the genome. Cell. 2008;132(2):311–322. 10.1016/j.cell.2007.12.014 18243105 PMC2669738

[34] Meyer LR, Zweig AS, Hinrichs AS, Karolchik D, Kuhn RM, Wong M, et al The UCSC Genome Browser database: extensions and updates 2013. Nucleic Acids Research. 2013;41(D1):D64–D69. 10.1093/nar/gks1048 23155063 PMC3531082

[35] Shao Z, Zhang Y, Yuan GC, Orkin SH, Waxman DJ. MAnorm: a robust model for quantitative comparison of ChIP-Seq data sets. Genome Biology. 2012;13(3):R16 10.1186/gb-2012-13-3-r16 22424423 PMC3439967

[36] Bryne JC, Valen E, Tang MHE, Marstrand T, Winther O, da Piedade I, et al JASPAR, the open access database of transcription factor-binding profiles: new content and tools in the 2008 update. Nucleic Acids Research. 2008;36(suppl 1):D102–D106. 10.1093/nar/gkm955 18006571 PMC2238834

[37] Kuhn RM, Karolchik D, Zweig AS, Trumbower H, Thomas DJ, Thakkapallayil A, et al The UCSC genome browser database: update 2007. Nucleic Acids Research. 2007;35(suppl 1):D668–D673. 10.1093/nar/gkl928 17142222 PMC1669757

[38] Voorhees EM. The TREC-8 Question Answering Track Report. In: TREC vol. 99; 1999 p. 77–82.

[39] Brill E, Lin JJ, Banko M, Dumais ST, Ng AY. Data-intensive question answering. In: TREC; 2001.

[40] Shao M, Yang Y, Guan J, Zhou S. Choosing appropriate models for protein-protein interaction networks: a comparison study. Briefings in Bioinformatics. 2013;p. bbt014. 10.1093/bib/bbt014 23515467

[41] Joachims T. Transductive inference for text classification using support vector machines. In: ICML vol. 99; 1999 p. 200–209.

[42] Marson A, Levine SS, Cole MF, Frampton GM, Brambrink T, Johnstone S, et al Connecting microRNA genes to the core transcriptional regulatory circuitry of embryonic stem cells. Cell. 2008;134(3):521–533. 10.1016/j.cell.2008.07.020 18692474 PMC2586071

[43] Toedling J, Servant N, Ciaudo C, Farinelli L, Voinnet O, Heard E, et al Deep-sequencing protocols influence the results obtained in small-RNA sequencing. PloS One. 2012;7(2):e32724 10.1371/journal.pone.0032724 22384282 PMC3287988

[44] Chen X, Xu H, Yuan P, Fang F, Huss M, Vega VB, et al Integration of external signaling pathways with the core transcriptional network in embryonic stem cells. Cell. 2008;133(6):1106–1117. 10.1016/j.cell.2008.04.043 18555785

[45] Gordon A. FASTX-toolkit. http://hannonlab.cshl.edu/fastx\_toolkit/index.html; 2010. [Online; accessed 22-September-2013].

[46] Langmead B, Trapnell C, Pop M, Salzberg SL. Ultrafast and memory-efficient alignment of short DNA sequences to the human genome. Genome Biology. 2009;10(3):R25 10.1186/gb-2009-10-3-r25 19261174 PMC2690996

[47] Friard O, Re A, Taverna D, De Bortoli M, Cor D. CircuitsDB: a database of mixed microR-NA/transcription factor feed-forward regulatory circuits in human and mouse. BMC Bioinformatics. 2010;11(1). 10.1186/1471-2105-11-435 20731828 PMC2936401

[48] Rhee HS, Pugh BF. Comprehensive genome-wide protein-DNA interactions detected at single-nucleotide resolution. Cell. 2011;147(6):1408–1419. 10.1016/j.cell.2011.11.013 22153082 PMC3243364

[49] Berger SL. Histone modifications in transcriptional regulation. Current Opinion in Genetics & Development. 2002;12(2):142–148. 10.1016/S0959-437X(02)00279-4 11893486

