# Supplementary material for: Identifying TF-MiRNA Regulatory Relationships Using Multiple Features
Source: PLoS One. 2015 Apr 29;10(4):e0125156. doi: 10.1371/journal.pone.0125156 (PMC4414601; doi:10.1371/journal.pone.0125156)
Supplement: S1 Text — Fig. A. The recall and removal rate of prediction on Klf4-miRNA relationships using protein-coding gene related positive data sets of transcription factor Klf4 and five-fold cross validation. In each panel, the x-axis denotes the parameter p of SVMlight. It ranges from 0.05 to 0.95 with a step size of 0.05. The y-axis denotes the recall (left panel) and the removal rate (right panel) of the prediction, respectively. Fig. B. The recall and removal rate of prediction on Oct4-miRNA relationships using protein-coding gene and miRNA gene related positive data sets of transcription factor Oct4 and five-fold cross validation. In each panel, the x-axis denotes the parameter p of SVMlight. It ranges from 0.05 to 0.95 with a step size of 0.05. The y-axis denotes the recall (left panel) and the removal rate (right panel) of the prediction, respectively. Fig. C. The recall and removal rate of prediction on Sox2-miRNA relationships using protein-coding gene and miRNA gene related positive data sets of transcription factor Sox2 and five-fold cross validation. In each panel, the x-axis denotes the parameter p of SVMlight. It ranges from 0.05 to 0.95 with a step size of 0.05. The y-axis denotes the recall (left panel) and the removal rate (right panel) of the prediction, respectively. Fig. D. The recall and removal rate of prediction on Tcf3-miRNA relationships using protein-coding gene and miRNA gene related positive data sets of transcription factor Tcf3 and five-fold cross validation. In each panel, the x-axis denotes the parameter p of SVMlight. It ranges from 0.05 to 0.95 with a step size of 0.05. The y-axis denotes the recall (left panel) and the removal rate (right panel) of the prediction, respectively. Table A. The genomic coordinates of the miRNA genes and their putative regulatory regions used in our study. Here Chr in the second column means the chromosome that the miRNA gene located in. Gstart and Gend mean the start and end coordinates of the miRNA gene, respectively. [file pone.0125156.s001.pdf]

Identifying TF-miRNA regulatory relationships using multiple features
(Supporting Figures and Tables)

Contents

1 Supporting Figures **1**

2 Supporting Tables **3**

1 Supporting Figures

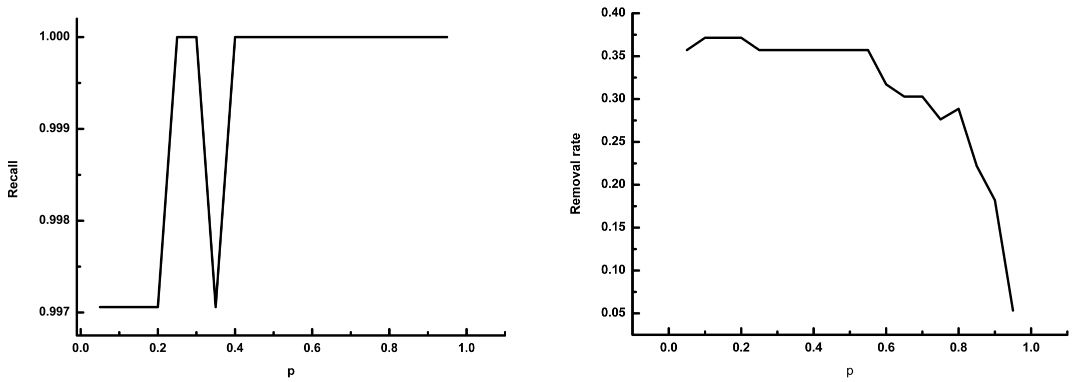

Figure A. The recall and removal rate of prediction on Klf4-miRNA relationships using protein-coding gene related positive data sets of transcription factor Klf4 and five-fold cross validation. In each panel, the x-axis denotes the parameter p of SVMlight. It ranges from 0.05 to 0.95 with a step size of 0.05.The y-axis denotes the recall (left panel) and the removal rate (right panel) of the prediction, respectively.

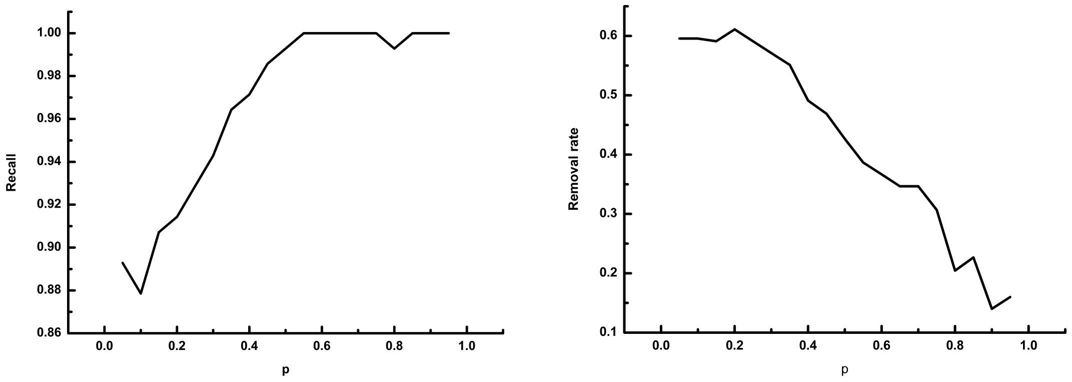

Figure B. The recall and removal rate of prediction on Oct4-miRNA relationships using protein-coding gene and miRNA gene related positive data sets of transcription factor Oct4 and five-fold cross validation. In each panel, the x-axis denotes the parameter p of SVMlight. It ranges from 0.05 to 0.95 with a step size of 0.05.The y-axis denotes the recall (left panel) and the removal rate (right panel) of the prediction, respectively.

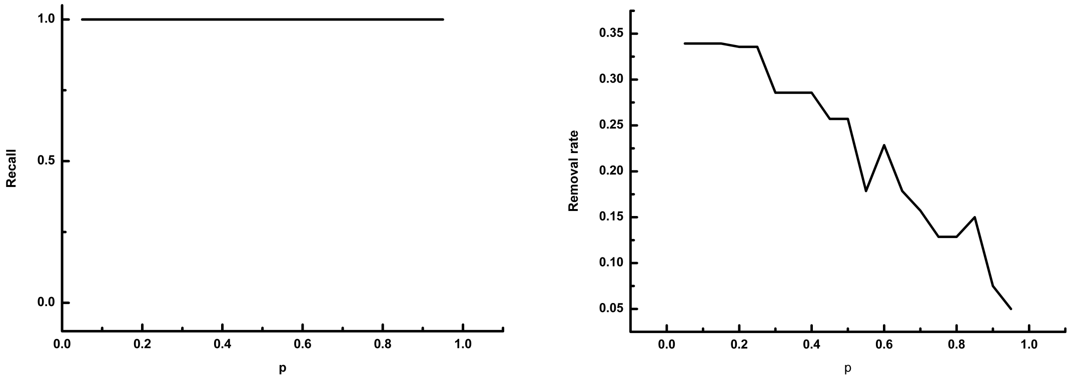

Figure C. The recall and removal rate of prediction on Sox2-miRNA relationships using protein-coding gene and miRNA gene related positive data sets of transcription factor Sox2 and five-fold cross validation. In each panel, the x-axis denotes the parameter p of SVMlight. It ranges from 0.05 to 0.95 with a step size of 0.05.The y-axis denotes the recall (left panel) and the removal rate (right panel) of the prediction, respectively.

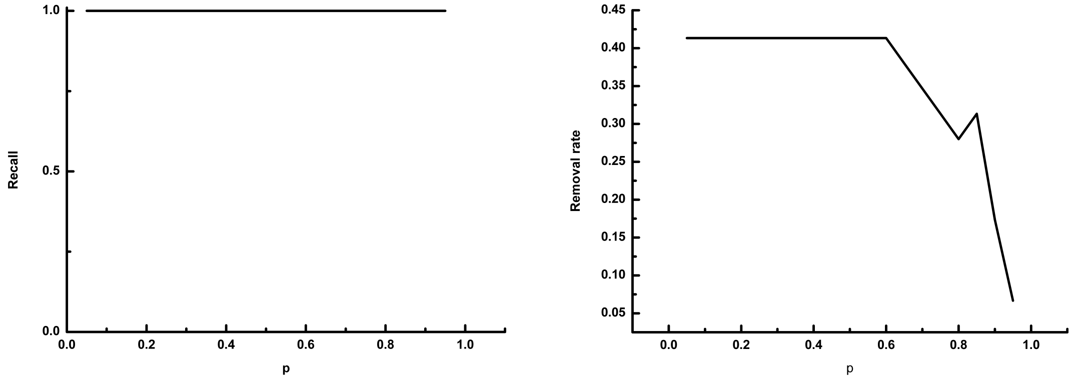

Figure D. The recall and removal rate of prediction on Tcf3-miRNA relationships using protein-coding gene and miRNA gene related positive data sets of transcription factor Tcf3 and five-fold cross validation. In each panel, the x-axis denotes the parameter *p* of SVMlight. It ranges from 0.05 to 0.95 with a step size of 0.05.The y-axis denotes the recall (left panel) and the removal rate (right panel) of the prediction, respectively.

2 Supporting Tables

Table A. The genomic coordinates of the miRNA genes and their putative regulatory regions used in our study. Here Chr in the second column means the chromosome that the miRNA gene located in. G_{start} and G_{end} mean the start and end coordinates of the miRNA gene, respectively. R_{start} and R_{end} mean the start and end coordinates of the putative regulatory regions of this miRNA gene, respectively. Please note that the start coordinate is always smaller than the end coordinate in this table, regardless of which strand the miRNA gene is located in.

miRNA gene	Chr	G_{start}	G_{end}	R_{start}	R_{end}
mmu-mir-206	chr1+	20679010	20679082	20392300	20679010
mmu-mir-133b	chr1+	20682769	20682887	20392300	20682769
mmu-mir-30a	chr1+	23272269	23272339	22572269	23272269
mmu-mir-30c-2	chr1+	23291701	23291784	22591701	23291701
mmu-mir-181a-1	chr1+	137966455	137966541	137266455	137966455
mmu-mir-181b-1	chr1+	137966639	137966718	137266639	137966639
mmu-mir-29b-2	chr1+	195037040	195037120	194961292	195037040
mmu-mir-29c	chr1+	195037547	195037634	194961292	195037547
mmu-mir-205	chr1-	193507463	193507530	193507530	194207530
mmu-mir-674	chr2+	117185127	117185226	117182277	117185127
mmu-mir-3090	chr2+	133564708	133564794	133562896	133564708
mmu-mir-130a	chr2-	84741115	84741178	84741178	84765359
mmu-mir-1953	chr2-	151967529	151967617	151967617	151969397
mmu-mir-298	chr2-	174267504	174267585	174267585	174281236
mmu-mir-190b	chr3+	90070020	90070099	90068347	90070020
mmu-mir-92b	chr3-	89227116	89227198	89227198	89234176
mmu-mir-34a	chr4+	150068454	150068555	149647899	150068454
mmu-mir-31	chr4-	88910557	88910662	88910662	89274472
mmu-mir-182	chr6-	30165918	30165992	30165992	30211289
mmu-mir-96	chr6-	30169446	30169551	30169551	30211289

continued on next page

continued from previous page

miRNA gene	Chr	G_{start}	G_{end}	R_{start}	R_{end}
mmu-mir-183	chr6-	30169668	30169737	30169737	30211289
mmu-mir-29a	chr6-	31062660	31062747	31062747	31366884
mmu-mir-148a	chr6-	51269812	51269910	51269910	51460434
mmu-mir-293	chr7+	3220343	3220422	3144992	3220343
mmu-mir-294	chr7+	3220641	3220724	3144992	3220641
mmu-mir-295	chr7+	3220773	3220841	3144992	3220773
mmu-mir-3099	chr7+	6803587	6803670	6707624	6803587
mmu-mir-1191	chr7+	27205536	27205655	27204320	27205536
mmu-mir-150	chr7+	45121757	45121821	45078503	45121757
mmu-mir-7a-2	chr7+	78888277	78888373	78792988	78888277
mmu-mir-344d-1	chr7-	61683124	61683192	61683192	61705849
mmu-mir-344b	chr7-	61790519	61790581	61790581	61943900
mmu-mir-344c	chr7-	61837311	61837402	61837402	61943900
mmu-mir-1965	chr7-	80152890	80153015	80153015	80227159
mmu-mir-210	chr7-	141221384	141221493	141221493	141263182
mmu-mir-1969	chr8+	70925525	70925618	70917529	70925525
mmu-mir-23a	chr8+	84208518	84208592	84172597	84208518
mmu-mir-27a	chr8+	84208672	84208758	84172597	84208672
mmu-mir-24-2	chr8+	84208815	84208921	84172597	84208815
mmu-mir-138-2	chr8+	94324311	94324381	94315066	94324311
mmu-mir-1199	chr8-	84011515	84011633	84011633	84030285
mmu-mir-181d	chr8-	84178716	84178787	84178787	84210941
mmu-mir-100	chr9+	41531425	41531504	41432079	41531425
mmu-mir-425	chr9+	108568777	108568861	108565566	108568777
mmu-mir-138-1	chr9+	122682876	122682974	122580647	122682876
mmu-mir-34c	chr9-	51103034	51103110	51103110	51270257
mmu-mir-184	chr9-	89802260	89802328	89802328	90091668
mmu-mir-135a-2	chr10-	92072086	92072185	92072185	92081745
mmu-mir-1251	chr10-	92137140	92137223	92137223	92171300
mmu-let-7i	chr10-	122985640	122985724	122985724	122992060
mmu-mir-216b	chr11+	28746191	28746276	28693276	28746191
mmu-mir-1934	chr11+	69663043	69663125	69656727	69663043
mmu-mir-497	chr11+	70234717	70234800	70216414	70234717
mmu-mir-144	chr11+	78073005	78073070	78060432	78073005
mmu-mir-365-2	chr11+	79726400	79726511	79694012	79726400
mmu-mir-196a-1	chr11+	96265164	96265265	96264484	96265164
mmu-mir-1932	chr11+	119390472	119390561	119381076	119390472
mmu-mir-146a	chr11-	43374397	43374461	43374461	43420247
mmu-mir-493	chr12+	109580233	109580315	109571729	109580233
mmu-mir-665	chr12+	109586314	109586407	109571729	109586314
mmu-mir-3070a	chr12+	109587943	109588031	109571729	109587943
mmu-mir-341	chr12+	109611500	109611595	109592851	109611500
mmu-mir-1188	chr12+	109611822	109611941	109592851	109611822
mmu-mir-370	chr12+	109618258	109618336	109592851	109618258
mmu-mir-1197	chr12+	109712317	109712436	109661711	109712317
mmu-mir-758	chr12+	109712810	109712890	109661711	109712810
mmu-mir-329	chr12+	109713481	109713577	109661711	109713481
mmu-mir-494	chr12+	109715318	109715402	109661711	109715318

continued on next page

continued from previous page

miRNA gene	Chr	G_{start}	G_{end}	R_{start}	R_{end}
mmu-mir-667	chr12+	109720006	109720097	109661711	109720006
mmu-mir-376c	chr12+	109722718	109722803	109661711	109722718
mmu-mir-300	chr12+	109724313	109724391	109661711	109724313
mmu-mir-381	chr12+	109726822	109726896	109661711	109726822
mmu-mir-487b	chr12+	109727333	109727414	109661711	109727333
mmu-mir-134	chr12+	109734139	109734209	109661711	109734139
mmu-mir-668	chr12+	109734732	109734797	109661711	109734732
mmu-mir-377	chr12+	109740510	109740577	109661711	109740510
mmu-mir-541	chr12+	109742409	109742498	109661711	109742409
mmu-mir-410	chr12+	109743715	109743795	109661711	109743715
mmu-mir-3072	chr12+	109747878	109747960	109661711	109747878
mmu-mir-203	chr12+	112130880	112130955	112127573	112130880
mmu-mir-1247	chr12-	110278048	110278129	110278129	110667688
mmu-mir-1983	chr13-	21896918	21897049	21897049	22002175
mmu-let-7d	chr13-	48536012	48536114	48536114	48577871
mmu-mir-3078	chr14+	64591185	64591271	64113751	64591185
mmu-mir-320	chr14+	70443510	70443591	70138206	70443510
mmu-mir-16-1	chr14-	61631880	61631972	61631972	62276228
mmu-mir-15a	chr14-	61632027	61632110	61632110	62276228
mmu-let-7b	chr15+	85707319	85707403	85593708	85707319
mmu-mir-30b	chr15-	68337415	68337510	68337510	69037510
mmu-mir-688	chr15-	102671792	102671866	102671866	102706776
mmu-mir-193b	chr16+	13449523	13449601	13417529	13449523
mmu-mir-365-1	chr16+	13453840	13453926	13417529	13453840
mmu-mir-484	chr16+	14159626	14159692	14101494	14159626
mmu-mir-155	chr16+	84714140	84714204	84685147	84714140
mmu-mir-1945	chr16-	11254368	11254445	11254445	11803720
mmu-mir-99b	chr17+	17830188	17830257	17734167	17830188
mmu-mir-7b	chr17+	56242988	56243098	56173955	56242988
mmu-mir-122	chr18+	65248861	65248926	65217826	65248861
mmu-mir-187	chr18-	24429110	24429170	24429170	24484961
mmu-mir-143	chr18-	61649196	61649258	61649258	61696836
mmu-mir-194-2	chr19+	6264643	6264728	6262304	6264643
mmu-mir-192	chr19+	6264844	6264932	6262304	6264844
mmu-mir-3475	chrX+	140310948	140311012	140106797	140310948
mmu-mir-221	chrX-	19146294	19146388	19146388	19846388
mmu-mir-222	chrX-	19146893	19146971	19146971	19846971
mmu-mir-92a-2	chrX-	52741838	52741928	52741928	52742562
mmu-mir-19b-2	chrX-	52741983	52742066	52742066	52742562
mmu-mir-20b	chrX-	52742113	52742192	52742192	52742562
mmu-mir-18b	chrX-	52742331	52742413	52742413	52742562
mmu-mir-450a-1	chrX-	53048154	53048244	53048244	53053111
mmu-mir-450a-2	chrX-	53048299	53048367	53048367	53053111
mmu-mir-351	chrX-	53053255	53053353	53053353	53070001
mmu-mir-503	chrX-	53053984	53054054	53054054	53070001
mmu-mir-322	chrX-	53054255	53054349	53054349	53070001
mmu-mir-743a	chrX-	66776757	66776818	66776818	67476818
mmu-mir-742	chrX-	66780373	66780437	66780437	67480437

continued on next page

continued from previous page					
miRNA gene	Chr	G_{start}	G_{end}	R_{start}	R_{end}
mmu-mir-741	chrX-	66796805	66796875	66796875	67496875
mmu-mir-463	chrX-	66799223	66799297	66799297	67499297
mmu-mir-880	chrX-	66800530	66800607	66800607	67500607
mmu-mir-881	chrX-	66801944	66802021	66802021	67502021
mmu-mir-470	chrX-	66813951	66814025	66814025	67514025
mmu-mir-201	chrX-	67988096	67988161	67988161	68667513
mmu-mir-547	chrX-	67988374	67988451	67988451	68667513

Table B. Final prediction result for the five TFs: Esrrb, Klf4, Oct4, Sox2, and Tcf3. The number in the parentheses behind the TF indicates the count of miRNAs regulated by this particular TF.

TF	miRNA				
Esrrb(57)	mmu-mir-295	mmu-mir-294	mmu-mir-34a	mmu-mir-1965	mmu-mir-377
	mmu-mir-30a	mmu-mir-27a	mmu-mir-298	mmu-mir-134	mmu-mir-1199
	mmu-mir-96	mmu-mir-30c-2	mmu-mir-365-1	mmu-mir-351	mmu-mir-365-2
	mmu-mir-181d	mmu-mir-29a	mmu-mir-210	mmu-mir-1983	mmu-mir-293
	mmu-mir-193b	mmu-mir-497	mmu-mir-31	mmu-mir-183	mmu-mir-1247
	mmu-mir-187	mmu-mir-133b	mmu-mir-182	mmu-mir-381	mmu-mir-1932
	mmu-mir-24-2	mmu-mir-688	mmu-mir-410	mmu-mir-668	mmu-mir-100
	mmu-mir-23a	mmu-mir-1945	mmu-mir-487b	mmu-mir-29b-2	mmu-mir-206
	mmu-mir-29c	mmu-mir-221	mmu-mir-222	mmu-mir-300	mmu-mir-320
	mmu-mir-138-1	mmu-mir-99b	mmu-mir-150	mmu-mir-92b	mmu-mir-130a
	mmu-mir-3078	mmu-mir-7b	mmu-mir-148a	mmu-mir-541	mmu-let-7b
	mmu-mir-3072	mmu-mir-3099			
Klf4(20)	mmu-mir-295	mmu-mir-294	mmu-mir-1934	mmu-mir-34a	mmu-mir-293
	mmu-mir-3078	mmu-mir-1965	mmu-mir-30b	mmu-mir-205	mmu-mir-99b
	mmu-mir-150	mmu-mir-7a-2	mmu-mir-1199	mmu-mir-1945	mmu-mir-1247
	mmu-mir-29b-2	mmu-mir-29c	mmu-mir-155	mmu-mir-320	mmu-mir-3099
Oct4(4)	mmu-mir-181d	mmu-mir-23a	mmu-mir-24-2	mmu-mir-27a	
Sox2(15)	mmu-mir-1983	mmu-mir-27a	mmu-mir-181a-1	mmu-mir-23a	mmu-mir-7b
	mmu-mir-7a-2	mmu-mir-150	mmu-mir-29a	mmu-mir-24-2	mmu-mir-221
	mmu-mir-181d	mmu-mir-133b	mmu-mir-206	mmu-mir-320	mmu-mir-3099
Tcf3(11)	mmu-mir-1983	mmu-mir-34a	mmu-mir-27a	mmu-mir-23a	mmu-mir-130a
	mmu-mir-3078	mmu-mir-201	mmu-mir-24-2	mmu-mir-221	mmu-mir-222
	mmu-mir-1247				
