# Supplementary material for: Identifying TF-MiRNA Regulatory Relationships Using Multiple Features
Source: PLoS One. 2015 Apr 29;10(4):e0125156. doi: 10.1371/journal.pone.0125156 (PMC4414601; doi:10.1371/journal.pone.0125156)
Supplement: S3 Text — (PDF) [file pone.0125156.s003.pdf]

Identifying TF-miRNA regulatory relationships using multiple features (Supporting Information)

1 Description of the feature values

This section gives a detailed explanation about the values of each feature used in our study. The categories of the features are consistent with that in the main text.

- **Statistical significance of peaks**: The values of this category of features are derived from the results of peak calling as can be seen in the provided files.
- **The location of peaks**: For DNAse-seq data, there are only two values for each peak. If the candidate peak is overlapped with DNAse-seq peaks, the feature value is 1. Otherwise, the feature value is 0. The distance feature is the actual distance (measured by bp) from peak summit to the start of the miRNA gene.
- **TFBS motifs**: We assigned each peak a motif score to indicate the level of similarity of the binding sites to the motif. We set the threshold to zero. If no hits with score ≥ 0.0 are found by the motif search program, the score is set to -1.
- **Evolutionary conservation**: Similar to the DNase-Seq feature, this is also a binary feature, if a peak has its homologous peak, this feature is set to one, otherwise, this feature is zero.

After obtaining all these features, for each miRNA, we used the mean reciprocal rank (MRR) to select the most "significant" peak to represent this miRNA, and used as the training example further for PU learning.
